# Innate Immunity and Immune Evasion by Enterovirus 71

**DOI:** 10.3390/v7122961

**Published:** 2015-12-14

**Authors:** Prabuddha S. Pathinayake, Alan C-Y. Hsu, Peter A.B. Wark

**Affiliations:** 1 Priority Research Centre for Asthma and Respiratory Disease, University of Newcastle and Hunter Medical Research Institute, New South Wales 2305, Australia; alan.hsu@newcastle.edu.au (A.C.Y.H.); peter.wark@hnehealth.nsw.gov.au (P.A.B.W.); 2 Department of Respiratory and Sleep Medicine, John Hunter Hospital, New South Wales 2305, Australia

**Keywords:** Enterovirus 71, innate immunity, Interferon antagonist, J0101

## Abstract

Enterovirus 71 (EV71) is a major infectious disease affecting millions of people worldwide and it is the main etiological agent for outbreaks of hand foot and mouth disease (HFMD). Infection is often associated with severe gastroenterological, pulmonary, and neurological diseases that are most prevalent in children. Currently, no effective vaccine or antiviral drugs exist against EV71 infection. A lack of knowledge on the molecular mechanisms of EV71 infection in the host and the virus-host interactions is a major constraint to developing specific antiviral strategies against this infection. Previous studies have identified and characterized the function of several viral proteins produced by EV71 that interact with the host innate immune proteins, including type I interferon signaling and microRNAs. These interactions eventually promote efficient viral replication and increased susceptibility to the disease. In this review we discuss the functions of EV71 viral proteins in the modulation of host innate immune responses to facilitate viral replication.

## 1. Introduction

Enteroviruses are a serious infectious disease and health concern across the Asia-Pacific region. In particular, EV71 causes sporadic outbreaks of hand-foot-and-mouth disease (HFMD), but may also lead to severe symptoms with prolonged illnesses and increased socio-economic burdens. EV71 belongs to the *Enterovirus A* species of the *Enterovirus* genus of the family *Picornaviridae.* This is a non-enveloped, single-strand positive sense RNA virus and shares similar genetic homology to coxsackievirus A16 (CAV16). EV71 also shares sequence homology with poliovirus 1M (58%), and with rhinovirus 1B (53%). The symptoms of central nerve system (CNS) complications caused by EV71 infection are frequently indistinguishable from poliomyelitis caused by poliovirus [1]. Infections with EV71 cause pulmonary dysfunctions and many other neurologic manifestations [2]. Because of its consecutive epidemics over past years in the Asia-Pacific region, it has attracted immense concern in global heath sectors. However, there is no effective treatment in humans. For the development of successful prevention and treatments against this virus, it is important to understand the mechanisms of infection of EV71 against the host immune system. Therefore, in this report we discuss the host-pathogen interaction elicited by EV71 proteins that antagonize the innate immune response and the potential that may exist to develop therapeutic treatments against EV infections.

The innate immune system provides an early phase defense against invading pathogens by recognizing diverse but conserved microbial moieties including viral RNAs, and then initiating an innate immune response that limits infection. The innate immune system response is not as specific as that of the adaptive immune system. However, it responds immediately to the presence of pathogens and can effectively limit infections, subsequently triggering appropriate signals that activate adaptive immune responses for clearance of infectious organisms [3,4]. Host cells recognize pathogens through the detection of pathogen-associated molecular patterns (PAMPs). Generally, host cells, including both non-immune cells (such as epithelial cells) or immune cells (such as macrophages), possess innate germline-encoded receptors for the identification of pathogenic materials. These receptors are known as pattern-recognition receptors (PRRs). Such PRRs can identify specific PAMPs such as bacterial lipopolysaccharide, flagellin, and bacterial or viral nucleic acids. PRRs are present both on the cell surface as well as within the cytoplasm or in the endosome and have evolved to recognize the presence of these foreign proteins [5,6].

In addition to foreign infectious materials, endogenous molecules released from stressed or dying cells are also able to induce innate immune signaling. These molecules have been named Danger Associated Molecular Patterns (DAMP). For instance, heat shock proteins (HSP), high-mobility group box 1 (HMGB 1), cytosolic RNAs, cytosolic DNAs including mitochondrial DNA released by damaged or stressed cells can be sensed by a range of PRRs and these signals also can trigger an innate and pro inflammatory response [7].

**Table 1:** Pattern-recognition receptors (PRRs) responsible for virus detection.

Pattern-Recognition Receptor (PRR)		Pathogen-Associated Molecular Patterns (PAMPs)
Toll Like Receptors		
TLR4		Viral Glycoproteins (RSV, MMTV)
TLR2		Viral Glycoproteins (Measles virus, HCMV, HSV-1, Epstein-Barr virus (EBV), RSV, LCMV)
TLR3		dsRNA (Reovirus, LCMV, MCMV, VSV, West Nile Virus, Punta Toro Virus)
TLR7 & 8		ssRNA (Influenza virus, VSV, Sendai virus, HIV, Coxsackievirus B)
TLR9		Viral DNA (HSV-1/-2, MCMV, EBV)
2. RIG-I-like receptors		
RIG-I		Uncapped 5′ppp ssRNA, short dsRNA (VSV, Rabies virus, Sendai virus, NDV, RSV, Influenza A&B, HCV, Japanese encephalitis virus, and Ebola virus)
MDA5		dsRNA (EV71, CVB, EMCV, theiler’s virus, and mengo virus)
3. Cytosolic DNA sensors		
IFI16	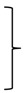	Viral DNA (HSV)
RNA polymerase III		
DAI		
LRRFIP1		
DDX9/36		
4. NOD Like Receptors		
NALP3		dsRNA

Viruses are obligatory intracellular microorganisms that must hijack the host’s protein synthesis machinery to replicate within the host cells. In mammalian cells there are three main classes of PRRs that recognize the virus PAMPs; namely toll-like receptors (TLRs), retinoic acid-inducible gene I (RIG-I) like receptors (RLRs) and nucleotide oligomerization domain (NOD) like receptors (NLRs) (Table 1).

Upon binding to viral DNAs/RNAs, TLRs (TLR3 and 7) and RLRs (RIG-I, melanoma-differentiation-associated (MDA5)), initiate signaling cascades that lead to the production of type I interferons (IFNs). These IFNs are essential in the early control of virus infection as they facilitate the induction of over 300 IFN-stimulated genes including MX1, OAS1, and PKR that degrade viral RNAs and induce apoptosis, resulting in an antiviral state in the microenvironment. TLR3/7 also mediates the induction of inflammatory cytokines including interleukin (IL)-6 that attracts neutrophils and macrophages to the site of infection. NLRs, particularly NLRP3 have also been shown to recognize viral RNAs via an unknown mechanism and facilitate the formation of inflammasome, which then mediates the activation and release of important inflammatory cytokine IL-1β [8,9].

Many viruses have evolved and developed different mechanisms to evade the innate immune system and promote viral replication inside the host cells. There have been extensive studies on the virology of EV71 and its interactions with the host innate immune responses. This review mainly focuses on the host-pathogen interactions elicited by the EV71 with a special focus on innate immunity.

### 1.1. Clinical Features of EV71 Infection

EV71 is a neurotropic virus that results in a clinical syndrome of hand foot and mouth disease (HFMD) and herpangina in children which tends to occurs in sporadic outbreaks [10]. In most cases it is a mild self-limiting illness. HFMD is characterized by mild fever, papular-vesicular rash on the palms or soles of the feet, as well as multiple oral ulcers. In a small number of affected cases it can result in more severe and life threatening disease. Infection with neurological complications can lead to life threatening disease; resulting in aspetic meningitis, acute generalized flaccid paralysis, and encephalitis. The encephalitis may affect the brainstem resulting in severe neurogenic induced pulmonary odema and severe cardiorespiratory symptoms [2].

Besides CNS complications, EV71 infections may also result in pulmonary disease. In severe cases, including those associated with brainstem encephalitis this may lead to pulmonary edema (PE) and subsequent cardio-pulmonary failure [11]. The etiology of which remains unclear is thought to be a consequence of neurogenic induced pulmonary edema as well as direct cardio/pulmonary toxicity.

In young children including infants it frequently causes symptoms consistent with an upper respiratory tract infection, as well as lower airway involvement, resulting in; exacerbations of bronchial asthma, bronchiolitis and pneumonia [12].

In experimental models, EV71 infected gerbils displayed pulmonary lesions characterized with interstitial pneumonia, pulmonary congestion and excessive lung haemorrhage along with neurologic symptoms [13]. Rhesus monkeys infected with EV71 demonstrated substantial inflammation in lungs. They developed severe interstitial pneumonia with alveolar damage, vessel hyperemia, high cellular infiltration around the terminal bronchioles and proliferation of lymphocyte follicles [14]. While the specific mechanisms of EV71 induced PE and pulmonary complications are not clear, the release of pro-inflammatory cytokines IL-6, IL-13, and IFN-γ have been shown to be important in the induction of mild pulmonary edema and pulmonary symptoms in EV71 infected mice [15].

### 1.2. Virus Infection and Innate Immune Response

Cells can detect various viral moieties such as genomic DNA, single-stranded RNA (ssRNA), double-stranded RNA (dsRNA), RNAs with 5′-triphosphate ends and viral proteins through different PRRs. Picornaviruses are mainly detected by MDA5 [16,17,18]. RIG-I and MDA5 both consist of two N-terminal caspase-recruitment domains (CARDs), a DExD/H box RNA helicase domain, and a C-terminal repressor domain (RD). The helicase domain and RD are important for the recognition of viral RNAs while CARD domain is important in the initiation of subsequent intracellular signaling cascades [19,20]. RIG-I recognizes 5′-triphosphate RNA and dsRNA during the RNA virus infection. MDA5 detects long dsRNA of >2 kb. It has been shown that the binding of RNAs by RIG-I and MDA5 is dependent on the length of RNA [21]. Once bound to viral RNAs, RIG-I and MDA5 undergo a conformational change that releases the CARD domain to interact with the same domain found on a downstream adaptor protein known as mitochondrial antiviral signaling (MAVS) located on the outer membrane of mitochondria [22,23]. MAVS associate with TNF-receptor-associated factor3 (TRAF3) and TRAF3 recruits and activates two IKK-related kinases, designated TANK-binding kinase 1 (TBK1) and inducible IkB kinase (IKK-i; also known as IKKe), which then phosphorylates IRF-3 and IRF-7 [24]. Phosphorylation of IRF-3 and IRF-7 induces the formation of homodimers and/or heterodimers that translocate into the nucleus and bind to IFN-stimulated response elements (ISREs), thereby resulting in the expression of type I IFN genes. NF-κB is also activated by MAVS via a FADD and caspase-8/-10-dependent pathway [25]. NF-κB plays an important role in innate immunity. It activates several components of the innate immune system such as pro-inflammatory cytokines, adhesion molecules, chemokine, acute phase proteins (e.g., Serum amyloid A), and inducible enzymes (e.g., iNOS and COX-2) *etc.* against pathogen invasions [26,27].

In addition to MDA5 and RIG-I, the TLR3 pathway also recognizes picornaviruses [28,29,30,31]. Upon binding to viral RNAs, TLR3 triggers signaling via TIR domain-containing adapter inducing IFN-β (TRIF), which associates with TRAF3 or TRAF6. TRIF also associates with receptor interacting protein 1 (RIP1), which then activates NF-κB and subsequently induces the release of inflammatory cytokines [32]. Once type I IFNs bind to Interferon receptors (IFNAR), they activate the receptor-associated protein tyrosine kinases Janus kinase 1 (JAK1) and tyrosine kinase2 (TYK2), which phosphorylate the latent cytoplasmic transcription factors signal transducer and activator of transcription 1 (STAT1) and STAT2 [33]. Phosphorylated STAT1 and STAT2 dimerize and translocate to the nucleus, where they assemble with IRF9 to form a trimolecular complex called IFN-stimulated gene factor 3 (ISGF3). ISGF3 binds to its cognate DNA sequences, which are known as IFN-stimulated response elements (ISREs) thereby directly activating the transcription of ISGs including protein kinase R (PKR) and ISG15 [34].

## 2. EV 71 Genome and Replication

**Figure 1:**
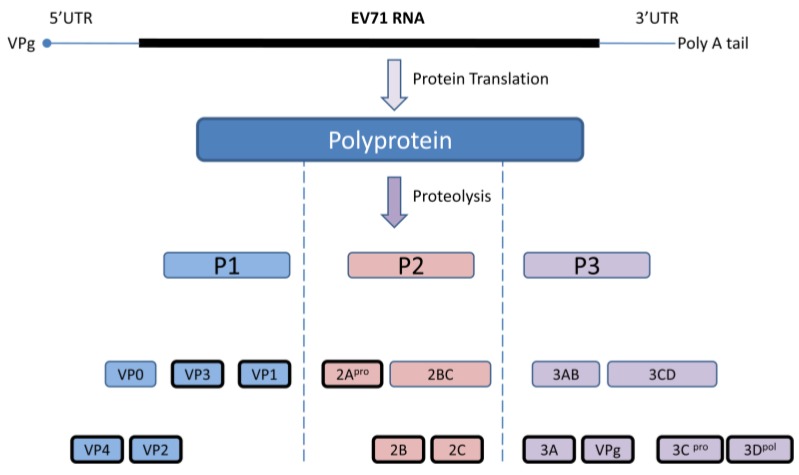
Diagrammatic representation of the EV71 genome and polyprotein products. Cap independent IRES driven protein translation produce a single polyprotein followed by proteolytic cleavage into partially processed products and eleven mature products. Virus encoded 3C and 2A proteases are accountable for this polyprotein processing at different stages.

EV 71 capsid consists of 60 identical protomers and each contains four different structural proteins; VP1-VP4. This viral capsid encloses single strand positive sense RNA that is approximately 7.4 kb in size. Viral particles are 20–30 nm in diameter and icosahedral in shape. EV71 primarily infects human through definite cell receptors named scavenger receptor B2 (hSCARB2), human P-selectin glycoprotein ligand-1(PSGL-1), and SA linked glycans [35,36]. Once the virus binds with the specific receptor on the host cell, the viral capsid undergoes structural changes and fuses with host plasma membrane, allowing viral RNA to be released into the cytoplasm. Subsequently, viral RNAs initiate cap-independent protein translation, polyprotein processing, and RNA replication for the production of new virions. The newly synthesized single viral polyprotein is cleaved by the viral 2Apro and 3Cpro into four structural (VP1, VP2, VP3, VP4) and seven non-structural (2A, 2B, 2C, 3A, 3C, and 3B, 3D) proteins in a proteolytic manner. During this proteolytic cleavage, it produces several viral precursors in addition to mature products (Figure 1). Both these precursors and mature proteins are actively involved in different viral processes including defense against host innate immunity [37].

## 3. Role of Type I IFNs on EV71 Infections

IFNs play a crucial role in mammalian responses to viral infections, controlling viral replication and spread [38]. The IFNs are classified into three groups, type I, II, and III based on their interaction with their receptors. Type I IFNs are produced upon viral infections and activate several antiviral effectors to form a defensive network against viral replication [39,40,41]. Certain subtypes of type I IFN (IFN-α4, IFN-α6, IFN-α14, and IFN-α16) have been identified as inhibitors of EV71 replication [42]. Specially, IFN-α14 was 20 times more effective than conventional IFN-α2a in preventing EV71 replication. Pre-treatment with IFN-α14 highly induced the expression of ISGs such as MX1, OAS1, and PKR and upon virus infection, it efficiently suppressed viral replication compared with the other subtypes of IFN-α [42]. However, these interferons can be only effective as a pre-treatment to control EV71 replication, Liu *et al.* [43] showed that pretreating mice with recombinant IFN-αA can protect against lethal EV71 infections, but post-treatment was much less effective. Moreover, administration of neutralizing antibody against IFN-α/β results in increased viral load and intensified virus-induced disease. The antiviral drug rupintrivir (EV71 3Cpro inhibitor) has limited efficacy against EV-71, however when combined with IFN-α, it was far more effective in suppression of EV71 replication [44]. Consistent with the observations of less effective post IFN treatment in controlling the EV71 infections in experimental setups, patients infected with EV71 have been shown to respond poorly to exogenous IFN treatments [45].

In addition to the type I interferons, type III interferons also have displayed a pivotal role in antiviral host defense. Type III interferons or lamda interferons (IFN-λ) comprise three isoforms, IFN-λ1 (IL-29), IFN-λ2 (IL-28A), and IFN-λ3 (IL-28B) [46,47,48]. IFN-λ2/3 are produced in both immune and non-immune cells in identical patterns regardless of the cell type and amplify the induction of IFN inducible genes [47]. Pott *et al.* [49] showed that type III interferons are crucial for antiviral immune defense in the intestinal epithelium. Since enteroviruses infections replicate in the gastro-intestinal tract, type III interferon may play a crucial role in controlling replication. It has been reported that, Coxsackievirus B3 (CVB3) replication is significantly reduced in type III interferon treated primary human pancreatic islets and that IFN-λ treatment induces several ISGs including MxA, RIG-I, and MDA5 [50,51]. However, IFN-λ did not exhibit significant protection against EMCV infections *in vivo* [47]. Therefore, the exact role of type III interferons in picornavirus infections still requires further elucidation.

Despite the effect of type I and III IFNs in controlling several other virus infections, EV71 has developed strategies to attenuate the antiviral immune responses by encompassing different mechanisms to down-regulate type I IFN production and promote viral replication. The mechanisms of inhibition in IFN signaling by EV71 are described in the next section.

## 4. 3C and 2A Proteases of EV71 Are the Main Antagonists of Type I IFN

**Figure 2:**
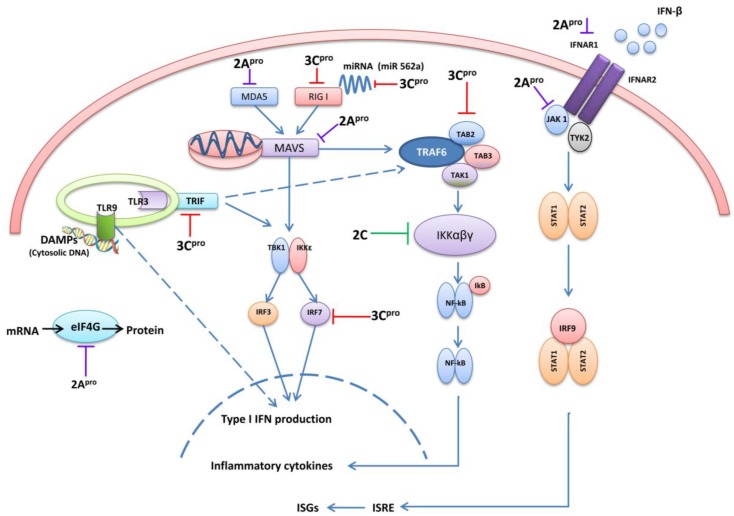
A graphical illustration of EV71 evasion strategies in cellular signaling pathways. EV71 3Cpro, 2Apro, and 2C are mainly involved in down-regulation of type I IFN, pro-inflammatory cytokines and ISGs induction at different stages. The interacting cellular signaling molecules with different viral proteins are indicated at each level. (**MDA5**: *Melanoma-differentiation-associated protein5*, **RIG-I**: *Retinoic acid-inducible gene 1*, **MAVS**: *Mitochondrial antiviral-signaling* protein, **TBK1**:*TANK-binding kinase 1, **IRF3/7**:Interferon Regulatory Factor 3/7*, **TRIF**: *TIR-domain-containing adapter-inducing interferon-β*, **TLR**: *Toll-like receptors*, **DAMPs**: *Danger associated molecular patterns*, **eIFG4**: *eukaryotic translation initiation factor 4G*, **TRAF6**: *TNF receptor-associated factor 6*, **TAK1**:*Transforming growth factor-β activated kinase 1*, **TAB2/3**: *TGF-β Activated Kinase 2/3*, **IFNAR**: *Interferon-α/β receptor*, **TAK1**: *Janus kinase 1*, **TYK2**: *Tyrosine Kinase 2*, **STAT1/2**: *Signal transducer and activator of transcription 1/2*, **ISG**: *interferon-stimulated genes*, **ISRE**: *Interferon*-*sensitive response element*, **IFNs**: *Interferons*).

3Cpro and 2Apro are essential for viral polyprotein processing. The crystal structure of EV71 3Cpro showed that it has a typical chymotrypsin-like fold that is common amongst 3Cpro proteins of other picornaviruses. Importantly it contains an important surface β-ribbon loop, at the base of which two important amino acid residues, Gly123 and His133, form a flexible hinge, and are important in the proteolytic activities of EV71 3Cpro [52]. In addition, it has been reported that 3Cpro has RNA binding ability. 3Cpro binds with the host RNA-dependent RNA polymerase that contains a nuclear localization sequence, allowing 3Cpro to enter into nuclei and facilitate in viral RNA replication [53,54]. The EV71 2A pro is a cysteine protease comprised of an N-terminal domain of a four-stranded anti-parallel β-sheet and a C-terminal domain of a six-stranded anti-parallel β-barrel with a tightly bound zinc atom [55]. Because of above characteristics, 3Cpro and 2Apro proteins play a dominant defensive role against host innate immunity by cleaving and binding to different antiviral signaling molecules (Figure 2).

### 4.1. 3C pro Interacts with RIG-I

MDA5 is the primary PRR for the recognition of picornavirus [16,17,18,56,57,58]. Previously, it was reported that MDA5 is cleaved during poliovirus infections in a proteasome and caspase-dependent manner [59]. Recently, Kuo *et al.* [56] explained the role of MDA5 in detecting EV71 RNA and subsequent IRF-3 activation using EV71-derived RNA as an agonist. Further, he demonstrated that overexpression of the MDA5 protein reverses the suppression of IRF3 activation caused by EV71 infection and EV71 infection enhances MDA5 degradation. However, the role of RLR upon EV71 infection further remains obscure.

Despite the importance of MDA5 in the recognition of picornaviruses, other studies have demonstrated RIG-I can be targeted for inhibition by several other picornaviruses (poliovirus, rhinoviruses, echovirus, and encephalomyocarditisvirus) [60]. Lei *et al.* [61] reported that EV71 3Cpro interacts with RIG-I and inhibits the type I IFNs production. Upon infection EV71 3Cpro directly binds with N terminal domain of RIG-I and impairs the interaction with the adaptor protein MAVS (also known as IPS-I /VISA/Cardif), leading to reduced activation of IRF3 and subsequent type I IFN production. The mutagenesis study of 3Cpro revealed that, this activity of EV71 3Cpro is specifically regulated by H40, KFRDI (amino acid 82–86) and VGK (amino acid 154–156) motifs which are indispensable for RNA binding and protease activity. Moreover, EV71 3Cpro substantially reduced the RIG-I-induced IFN-β promoter assay in a dose-dependent manner whilst it didnot affect the MDA-5-mediated IFN-β activity [61].

The exact role of EV71 3Cpro binding with RIG-I is unclear. There is no evidence to indicate RIG-I activation during EV71 infection. Likewise, RIG-I knockdown using siRNA did not prevent IRF3 activation upon EV71 viral RNA transfection but MDA5 knockdown drastically reduced the activation of IRF3 [56]. However, rhinovirus (RV1B) infections (a picornavirus) induced a robust RIG-I activation in human bronchial epithelial cells (HBECs). Upon RV1B infections in HBECs, RIG-I and MDA5 mRNA levels induced at 8 h post infection (hpi), peaked by 18 hpi and remained at the same level until 48 hpi. Consistently, both RIG-I and MDA5 protein levels showed a clear induction time dependent manner. Knockdown of RIG-I in HBECs reduced the IFN-β production upon RV1B infection but not type III IFN induction where as MDA5 knockdown impaired both type I and III IFNs [62]. In contrast to the RIG-I induction in HBECs in *in vitro*, *in vivo* RV challenge in bronchial biopsies did not show any RIG-I induction at four days post RV16 infection. However, the limitation of this experiment is that RIG-I/MDA5 was only measured at one time point after infection. In addition, they used different strains of virus (RV1B for HBEC and RV16 for biopsy) for different experiments. This therefore makes comparisons difficult. Therefore, further advanced researches are required to confirm the importance of RIG-I during picornavirus infections.

### 4.2. The Role of TRIF and Cleavage by 3Cpro

TLR3 is located on the endosomal membrane and recognizes mainly the viral double strand RNA (dsRNA). dsRNA-bound-TLR3 recruits TIR domain-containing adaptor inducing IFN-β (TRIF or TICAM1) which then activates IRF3 and NF-κB and initiates an antiviral state and inflammatory responses in the infected cells. TLR3/TICAM1 pathway is also important in immune responses against picornavirus infections. During poliovirus infection, human PVR transgenic TICAM-1^−/−^ mice showed a higher susceptibility to infection compared with wild type and MAVS^−/−^ transgenic mice. In addition, the type I IFN mRNA levels in splenic dendritic cells were lowest in TICAM-1^−/−^ transgenic mice over the other groups [28]. Another recent study reveals that MDA5, TLR3, and MyD88 mediated pathways contribute to antiviral response to poliovirus infection but TLR3mediated response is pivotal [29].

Abe *et al.* [29] explained the role of MDA5 in the detection of poliovirus (PV) in IFN primed primary murine kidney cells. They observed a significant inhibition of type I IFN induction upon PV infection (MOI 10) in MDA^−/−^/RIG-I^+/−^ cells but not in RIG-I^−/−^/MDA5^+/−^ cells. However, based on *invivo* studies, they concluded TLR3 was the key PRR for PV recognition. Additionally, they described that PV replication in non-neural tissues was independent of RLRs but mostly dependent on TLR3. Viral load in non-neural tissues of poliovirus infected TRIF^−/−^ mice showed a notably higher level compared with wild type mice. However, there was no significant difference in neural tissues from either TRIF^−/−^ mice or wild type mice. Therefore, it was suggested that the TLR3-TRIF-mediated pathway plays an important role in non-neural tissues to inhibit viral replication before viral invasion of the central nervous system (CNS) occurs rather than after invasion and that this response plays an important role in preventing CNS infection [29]. In addition, TLR3 elicited a role in controlling coxsackievirus and encephalomyocarditis virus infections [30,31]. Despite the importance of TLR3 pathway in picornavirus infection, Lei *et al.* [63] demonstrated that EV71 3Cpro can cleave TRIF at amino acid Q312-S313 and attenuate IRF3 and NF-κB mediated immune responses, including ISG56, ISG54, and IFN-β inductions. The TRIF cleavage upon EV71 infection became evident at 12–24 h post infection, however, poly I.C. induced ISGs inhibition by EV71 appeared at early time points (6–16 h post infection) [63]. Therefore, it is difficult to conclude that TRIF cleavage directly leads to ISGs inhibition. As 3Cpro protein appeared at 4 h post infection it may interact with TRIF at early time points although the TRFI protein expression is too small to be detected. Additionally, 3Cpro can interact with other cellular signaling proteins such as RIG-I, IRF7, IRF9 *etc.* which are crucial for ISGs induction and attenuates their function to inhibit the ISGs. Besides 3Cpro, EV71 viral protease 2Apro also can cleave several other cellular signaling molecules and inhibit ISGs induction. Therefore, further studies clearly are required to address these contradictory observations.

### 4.3. 3C pro Cleaves Interferon Regulatory Factor 7 (IRF7) and IRF9

IRF7 is an important transcription factor that induces type I IFNs productions. Once IRF7 receives signals from various PRRs (RIG-I, MDA5, TLR3, TLR7, TLR8, TLR9), it is phosphorylated and translocated into nucleus to induce type I IFNs production. Moreover, IRF3 acts on the priming stage of type I IFNs production while IRF7 is involved in the amplifying stage and resulting in a positive feedback to the initial response [64,65,66,67]. EV71 3Cpro has also been shown to cleave IRF7 and it impairs the induction of type I IFNs, and subsequent production of ISGs such as ISG56 in a dose-dependent manner. This cleavage occurs independently of caspases, proteasome, lysosome, and autophagy activities but merely a viral proteolytic event of 3Cpro [64]. Interferon Regulatory Factor 9 (IRF9) plays a crucial role in activating the transcription of ISGs. Hence, IRF9 invokes a decisive role on activating the anti-viral state in cells. It is reported 3Cpro also binds and degrades IRF9, although the exact molecular mechanisms of such interaction remain unclear. The IRF9 cleavage becomes evident at 5 h post infection onwards in cell based assays. The IRF9 cleavage activity was confirmed by enzyme assays using *E. coli* expressed recombinant IRF9 and purified recombinant 3Cpro. However, the point mutated recombinant 3Cpro (3CM) could not cleave IRF9 at the same condition in enzyme based assays [44].

### 4.4. 3Cpro Cleaves the TAK1/TAB1/TAB2/TAB3 Complex

Transforming growth factor-β activated kinase 1 (TAK1) is a member of the mitogen-activated protein kinase kinasekinase (MAP3K) family. It can be activated by TLR ligands, tumor necrosis factor alpha (TNF-α), and interleukin-1β (IL-1β). During infection, TAK1 forms a TAK1/TAB1/TAB2/TAB3 complex which then activates IKK αβγ, p38, and c-Jun N-terminal kinase (JNK) and triggers downstream NF-κB promoter activation [68,69]. EV71 3Cpro has been shown to directly target the TAK1/TAB1/TAB2/TAB3 complex. 3Cpro directly associates with and degrades TAK1 and TAB2 by proteolytic cleavage. Cleavage of TAK1/TAB1 then results in reduced activation of NF-κB and downstream induction of inflammatory cytokines. Interestingly, over-expression of TAB2 inhibits the EV71 replication by 50% while TAK1, TAB1, and TAB3 over expression elicited very little effect [70]. However, the distinct functions of each molecule in the TAK complex related to EV71 infection has yet to be further elucidated.

### 4.5. 3Cpro Cleaves CSTF-64

Cleavage stimulation factor (CSTF)-64 is a nuclear protein involved in the 3′ end cleavage and polyadenylation of pre-mRNAs, and therefore results in the maturation of mRNAs [71]. 3Cpro was shown to be translocated into nucleus and cleaves CSTF-64 protein and attenuate host protein transcriptions [72]. However, poly (A) synthesis of EV71 viral RNAs is not affected. A similar mechanism to inhibit cellular 3′-end pre-mRNA processing was also found during influenza A virus infection by the non-structural (NS)-1 protein. This inhibition of CSTF suppresses overall host protein synthesis and promotes virus replication [73]. Further investigates are required to understand the molecular mechanisms of EV71-mediated inhibition of host cellular mRNA processing.

### 4.6. 3Cpro Manipulates Cellular Micro RNAs

MicroRNAs (miRNAs) are highly conserved small non-coding RNA oligonucleotides and able to suppress mRNA translation by binding to the 3′ untranslated region (UTR) of target mRNAs. Thus, miRNAs are important in regulation of diverse biological processes including host immune responses. They can act as either inducers or suppressers of the host immune system [74]. EV71 infections have been shown to increase the levels of miRNAs and promote virus replication. In cells with depleted miRNA (mRNA depletion was carried out by the knockdown of DiGeorge syndrome chromosomal region 8 (DGCR8) gene, which is an important co-factor for cellular mRNA biogenesis) EV71 replication was found to be substantially reduced compared to controls cells. Depletion of miRNAs up-regulated TLR signaling, RLR signaling, NOD signaling and type 1 IFN signaling. Moreover, microarray and qPCR analysis showed that miR-548 is a potential candidate that may participate in the inhibition of host antiviral responses during EV71 infection [75].

Another study discovered that miR-141 is up-regulated upon infections with picornaviruses including EV71, poliovirus 3 (PV3) or coxsackievirusB3 (CVB3). miR-141 was shown to facilitate EV71 viral propagation by expediting a shift from cap-dependent to cap independent protein translation [76]. One of the targets of miR-141 is eIF4E, which is an essential component in cap-dependent translation. Inhibition of miR-141 by a specific antagomiR was able to restore host eIF4E expression and delay the occurrence of infection-induced cytopathic effect (CPE), and reduce viral replication. Additionally, a transcription factor, Early Growth Response 1 (EGR1), a nuclear protein required for differentiation and mitogenesis [77], was identified via microarray analysis to be highly induced (100 fold) by EV71 infection. Interestingly, two potential EGR1 binding sites were identified in the putative regulatory element of miR-141 and both sites are essential for maximal expression of miR-141 [76].

miR-146a is another miRNA that has been shown to regulate immune responses. The validated targets of miR-146a include IRAK1 and TRAF6, and EV71 infection has been shown to up-regulate the induction of this miRNA and suppress IRAK1 and TRAF6 expression, leading to reduced IFN-β production. Notably, miR-146a^−/−^ mice exhibited a high survival rate and lower clinical symptoms compared with wild type mice against pathogenic EV71 infection [78]. Similarly, miR-562a was shown to be increased during virus infections (VSV), in an IRF3/IRF7-dependent manner. The elevated level of miR-562a leads to enhanced RIG-I-mediated IFN-β production by suppressing the expression of cylindromatosis (CYLD) deubiqutinase activity, a negative regulator of RIG-I K63-linked ubiqutination. In contrast, the level of induction of miR-562a by EV71 infection is significantly low with minimal induction of IFN-β, and it is revealed that 3Cpro may attribute to this function as it cleaves IRF7 [79].

miRNAs are an important part of innate immune regulations, however, their functions may be nonspecific and specific targeting of miRNAs may have various off-target effects. Therefore, comprehensive investigations are required to analysis their functions before using them as potential antiviral therapeutic agents.

## 5. EV71 2Apro Disrupts IFN Signaling

EV71 2A is a cysteine protease responsible for cleavage of viral polypeptide to generate capsid protein precursor, 3C, 3D, and itself [80,81]. EV71 2A also cleaves several host cellular proteins, mostly related to type I IFNs signaling.

### 5.1. 2Apro Cleaves MAVS

Given the central role of MAVS in IFN signaling, MAVS is a target for suppression by several viruses. For instance, HCV-derived NS3/4A protease binds with and cleaves MAVS at Cys508 [82,83]. The protease precursor 3A/B/C/m of HAV has also been shown to cleave MAVS at Gln428 and impair the induction of type I IFNs [84]. Rhinovirus cleaves MAVS by its 2Apro and 3Cpro proteases [85]. CVB3 cleaves MAVS at Gln148 [86]. Interestingly, EV71 was also reported to cleave MAVS in proteolytic manner. The viral protease 2Apro cleaves MAVS at Gly209, Gly251, and Gly265. The cleaved products were detected in the cytosolic proteins by differential centrifugation followed by immunoblot and showed MAVS dissociation from mitochondria to the cytoplasm. Additionally, EV71 VP1 was shown to be co-localized with mitochondria and it is suggested that EV71 viral replication cycle involves the mitochondria, and viral proteins expressed during EV71 propagation may cause mitochondria dysfunction and induce MAVS cleavage. Consequently, it could abolish the IRF3 phosphorylation, type I IFN production and increase the virus propagation [87]. Notably, this action is independent of apoptosis or proteasome degradation.

### 5.2. 2Apro Cleaves MDA5

MDA5 seems to be the key receptor for detecting picornaviruses [18,56,88]. Upon viral RNA recognition, MDA5 interacts with MAVS and signals for the induction of type I IFNs. EVs including CVB3 and poliovirus 2Apro have been shown to cleave MDA5 during infections and CVB3 mediated MDA5 cleavage resulted in the attenuation of IFN production [59,89]. In contrast, Barral *et al.* [59] demonstrated that MDA5 cleavage by poliovirus infection is proteasome and caspase dependent. However, it is likely that IFN priming of cells before infection can upregulate ISGs including MDA5 and this can lead to MDA5 cleavage by proteasome and caspase in a dependent manner whereas in naïve cells viral proteases facilitate this cleavage [89]. Similar to the other enteroviruses, EV71 2Apro has also been shown to cleave MDA5, which is the foremost molecule for antiviral signal induction. This disruption of MDA5 by enterovirus 2Apro diminishes the IRF3 activation and down-regulates type I IFN production [89].

### 5.3. 2Apro Reduces Interferon Receptor I (IFNAR I) Expression

Upon virus infections, type I IFNs bind with IFNARs and activate JAK1and TYK2 which phosphorylate STAT1and STAT2, leading to the transcription of ISGs [34]. ISGs such as PKR and ISG15 then suppress viral replications. As discussed previously exogenous IFN treatments have limited effectiveness in EV71 infected patients. This is crucial for children with EV-mediated neurological manifestations. However, the molecular mechanisms underpinning this insensitivity to IFNs remain unclear. Recently, Lu *et al.* [45] reported that EV71 infection can significantly reduce IFNAR1 protein expression and impede the IFN response. EV71 infections in RD cells led to increases in IFN-β mRNA in a multiplicity of infection (MOI) dependent manner, but failed to induce any ISGs to a considerable level. In addition, phosphorylation of p-STAT1, p-STAT2, and Tyk2 and Jak1 were all decreased following infection. It was then shown that EV71 2Apro alone could degrade IFNAR1, p-Tyk2, and p-STAT1 levels whereas 2A mutant with defective protease activity was unable to show IFN antagonism. While exogenous IFN-α treatment alone could up-regulate these protein levels, EV71 infection in IFN-α pre-treated cells could still impair ISG inductions and increases virus replication, consistent with the ability of reduced IFNAR1 expression by EV71. Another study showed that 2Apro is essential for EV replication in IFN pre-treated cells [90]. As cells with decreased expression of IFN receptors are unable to respond to exogenous IFNs [91], this explains why post-treatment had limited effect on EV71 replication.

In contrast, Liu *et al.* [92] demonstrated that EV71 did not significantly alter IFNAR1 expression but it did inhibit the phosphorylation of JAK1 and Tyk2 independent of EV71 2Apro and 3Cpro inhibitory activity in IFN signaling. However, these controversial results have to be more clearly defined through comprehensive and repetitive experiments.

### 5.4. 2Apro Induces eIF4G Cleavage

eIF4G is a protein involved in eukaryotic protein translation, which is essential for the function of the host cell. However, several picornaviruses have been shown to cleave eIF4G during infections [93,94,95]. Similarly, EV71 2Apro also cleaves eIF4G and induces cellular apoptosis showing nuclear alterations, DNA fragmentations and PARP degradation. [81]. As picornaviral genome has the ability to translate viral proteins in a cap-independent manner, cleavage of eIF4G does not impair viral replication. However, it may affect the cellular protein translations including cellular anti-viral proteins. Therefore, the cleavage of eIF4G by picornaviruses including EV71 may be a strategy to shut off the host defense mechanism against virus infections. However, proper experimental evidence is required to prove this phenomenon.

## 6. EV71 2Cpro Inhibits NF-κB Activation

NF-κB is a protein complex that controls several gene transcriptions related to immune responses, cell proliferation, differentiation, *etc.* Additionally, NF-κB plays a fundamental role in host antiviral responses by inducing the expression of type I IFNs, pro-inflammatory cytokines [96,97,98]. However, EV71 2Cpro protein has been reported to inhibit NF-κB activation and enhances the virus replication. 2Cpro protein directly interacts with kinase domain of IKKβ and IκBα, and inhibits its phosphorylation. This reduces the activation of NF-κB and leads to accelerate viral replication [99].

## 7. Potential Antiviral Therapeutics

Currently, there are no effective antiviral drugs active against EV71 infection. Rupintrivir (AG7088) is a 3Cpro inhibitor that has been evaluated as a potent antiviral drug against EV71 infection. Previous studies revealed that small concentrations of rupintrivir (0.1 mg/Kg) can protect suckling mice from virulent EV71 induced paralysis and death [100]. However, it has to be further evaluated through rigorous clinical trials. There are several other drugs that have also been investigated and evaluated [101], although proven ineffective in the suppression of EV71 infection. Therefore, screening of natural and synthetic compounds that show strong antiviral properties specifically against 2A and 3C functions are important.

Additionally, exploring the role of TLR agonists as therapeutic agents against EV71 infection maybe worthwhile. Notably, TLR3, TLR7, and TLR9 are considered the best potential targets for antiviral therapy [102]. A variety of TLR agonists have been shown to inhibit hepatitis C and hepatitis B viral infection [103,104,105]. Intranasal administration of a TLR7/8 agonist significantly reduced the viral titer in an influenza virus infected rat model [106]. Additionally, there are some reports showing immune/antiviral enhanced responses from TLR3, TLR9, and TLR4 agonists in mice models of influenza infection [107,108,109]. A TLR8 agonist has shown to activate human neonatal antigen presenting cells and work as an immune-stimulant [110]. Recently it has been reported that TLR9 mediated protection in EV71 infected mice is due to the release of danger-associated molecular patterns (DAMPs). The endogenous DNA of apoptotic cells in the supernatant of EV71 infected cells is capable of activating TLR9 signaling and release of IFN-α, IFN-γ, MCP-1, TNF-α, IL-6, and IL-10 [111]. Therefore, TLR agonist may play an important role in the host defense against EV71 infection.

Previously, RNA aptamers have been successfully used to control the FMDV replications *in vitro* [112]. We demonstrated the ability of using DNA aptamers to suppress the influenza virus NS1 induced interferon antagonism [113]. Therefore, evaluation of DNA/RNA aptamer therapeutics against EV71 infection may also be valuable.

However, a major issue regarding EV71 research is the lack of an appropriate animal model susceptible to EV71 infection. At the moment the only available models rely upon a highly concentrated mouse-adapted viruses or the use of neonatal mice for *in vivo* infection experiments. As neonatal mice have an immature immune system they are highly susceptible to the virus. However, several practical difficulties are associated with the use of neonatal mice. One major concern is that, the immaturity of their immune system greatly limits the generalizability of their responses to childhood human infection. Additionally, it is difficult to use them for vaccine studies and antiviral drug therapies. However, recent studies have shown that IFN receptor knockout mice (AG129) have a higher susceptibility to non-mouse adapted EV71 infection. Some other studies have revealed that IFNs have a role in tissue tropism in EV infections [114,115]. Therefore, tissues not responsive to exogenous IFN treatment may promote viral replication and high pathogenicity in mice. Additionally, hSCARB2 receptor transgenic mice have displayed a higher vulnerability to EV71 infection since over-expression of hSCARB2 enhances the viral entry into different tissues and displays a high pathogenicity.

## 8. Discussion

Although poliomyelitis has been mostly eradicated worldwide, several outbreaks of EV71 related poliomyelitis-like neurological disorder have emerged especially in the Asia Pacific region in the last few decades. After the first isolation of this virus in California, in 1969, outbreaks have been reported in Australia, Sweden, Japan, Bulgaria, Hungary, Malaysia, Taiwan, Kenya, Singapore, Korea, Vietnam, Brunei, and China. Major outbreaks were reported in Sarawak-Malaysia (1997), Taiwan (1998), Singapore (2000), and China (2008) with many fatal cases [2]. Because of these fatal outbreaks, EV71 has become a global threat and has attracted the attention of health care agencies to produce effective treatments and better understand the pathogenesis of these infections, and understand their ability to evade host immune responses.

Effective innate immune activation is an important factor in controlling viral infections. The long co-evolution of EVs in humans has promoted the development of effective immune evasion strategies that effectively subvert host immunity. As with other picornaviruses, EV71 also encodes its own proteases; 3Cpro and 2Apro for viral polyprotein processing. The proteolytic nature of these viral proteins has evolved to target and cleave several intracellular signaling proteins to attenuate their function and promote viral replication (Figure 2). In addition to the aforementioned viral proteases and their IFN antagonistic properties, viral proteins including EV71 2B, 3A, and 3D have also been shown to have moderate inhibitory function of IFN-β promoter activity. Other viral proteins VP1, VP2, VP3, VP4, 2AB, 2BC, and 3AB do not have such inhibitory functions [61]. Nevertheless, the function of 2B, 2A, and 3D on innate immune signaling has not been comprehensively evaluated.

There have been many studies conducted to evaluate the host-pathogen interactions of EV71. However, some findings are controversial. For instance, the PRRs responsible for EV71 detection are still in discussion. Despite the involvement of MDA5 pathway in EV71 detection, the purpose of interaction of RIG-I with 3Cpro is not clear. Therefore, further studies are vital to confirm their roles in viral pathogenesis. Recently the importance of microRNAs in signaling and the host immune response has been investigated. For example miR-146 has been shown to down-regulate IRAK1 and TRAF6 expression, and inhibits IFN production. miR-146a^−/−^ mice showed increased IFN production [78]. However, as EV71 proteases disrupt multiple signaling proteins including RLRs, JAK-STAT pathway and inhibit ISG inductions, how miR-146a^−/−^ mice survived against infections is still unclear. Further investigations are required to determine the molecular mechanism in infections using several serotypes of EV71 and other enteroviruses.

Ultimately the development of a novel vaccine, which is capable of inducing long lasting cross protective immunity against multiple strains of EV71 and other major causative agents of HFMD such as CVB16 is required. However, due to frequent unpredictable switching of genotypes and subgenotypes of EV71, production of such an effective vaccine is challenging. Understanding the molecular interactions between EV71 and the host immune responses, their behavioral characters and identifying novel therapeutic targets including virus or host proteins is essential. Despite the fact that, there is an extensive current knowledge of the virology of picornaviruses, further structural and functional characterizations of EV71 proteins are needed to understand how these viral virulence factors interact with the human immune system. This would provide novel insights into the development of potential therapeutic options against EV infections.

## References

[1] Brown B.A., Pallansch M.A. (1995). Complete nucleotide sequence of enterovirus 71 is distinct from poliovirus. Virus Res..

[2] Solomon T., Lewthwaite P., Perera D., Cardosa M.J., McMinn P., Ooi M.H. (2010). Virology, epidemiology, pathogenesis, and control of enterovirus 71. Lancet Infect. Dis..

[3] Schenten D., Medzhitov R. (2011). The control of adaptive immune responses by the innate immune system. Adv. Immunol..

[4] Kumar H., Kawai T., Akira S. (2011). Pathogen recognition by the innate immune system. Int. Rev. Immunol..

[5] Kumagai Y., Takeuchi O., Akira S. (2008). Pathogen recognition by innate receptors. J. Infect. Chemother..

[6] Kawai T., Akira S. (2011). Toll-like receptors and their crosstalk with other innate receptors in infection and immunity. Immunity.

[7] Land W.G. (2015). The Role of Damage-Associated Molecular Patterns in Human Diseases: Part I—Promoting inflammation and immunity. Sultan Qaboos Univ. Med. J..

[8] Takeuchi O., Akira S. (2009). Innate immunity to virus infection. Immunol. Rev..

[9] Petrilli V., Dostert C., Muruve D.A., Tschopp J. (2007). The inflammasome: A danger sensing complex triggering innate immunity. Curr. Opin. Immunol..

[10] Ooi M.H., Wong S.C., Lewthwaite P., Cardosa M.J., Solomon T. (2010). Clinical features, diagnosis, and management of enterovirus 71. Lancet Neurol..

[11] Liu C.C., Tseng H.W., Wang S.M., Wang J.R., Su I.J. (2000). An outbreak of enterovirus 71 infection in Taiwan, 1998: Epidemiologic and clinical manifestations. J. Clin. Virol..

[12] Merovitz L., Demers A.M., Newby D., McDonald J. (2000). Enterovirus 71 infections at a Canadian center. Pediatr. Infect. Dis. J..

[13] Xu F., Yao P.P., Xia Y., Qian L., Yang Z.N., Xie R.H., Sun Y.S., Lu H.J., Miao Z.P., Li C. (2015). Enterovirus 71 infection causes severe pulmonary lesions in gerbils, merionesunguiculatus, which can be prevented by passive immunization with specific antisera. PLoS ONE.

[14] Zhang Y., Cui W., Liu L., Wang J., Zhao H., Liao Y., Na R., Dong C., Wang L., Xie Z. (2011). Pathogenesis study of enterovirus 71 infection in rhesus monkeys. Lab. Investig..

[15] Huang S.W., Lee Y.P., Hung Y.T., Lin C.H., Chuang J.I., Lei H.Y., Su I.J., Yu C.K. (2011). Exogenous interleukin-6, interleukin-13, and interferon-gamma provoke pulmonary abnormality with mild edema in enterovirus 71-infected mice. Respir. Res..

[16] Hornung V., Ellegast J., Kim S., Brzozka K., Jung A., Kato H., Poeck H., Akira S., Conzelmann K.K., Schlee M. (2006). 5′-Triphosphate RNA is the ligand for RIG-I. Science.

[17] Kato H., Takeuchi O., Sato S., Yoneyama M., Yamamoto M., Matsui K., Uematsu S., Jung A., Kawai T., Ishii K.J. (2006). Differential roles of MDA5 and RIG-I helicases in the recognition of RNA viruses. Nature.

[18] Feng Q., Hato S.V., Langereis M.A., Zoll J., Virgen-Slane R., Peisley A., Hur S., Semler B.L., van Rij R.P., van Kuppeveld F.J. (2012). MDA5 detects the double-stranded RNA replicative form in picornavirus-infected cells. Cell Rep..

[19] Yoneyama M., Kikuchi M., Natsukawa T., Shinobu N., Imaizumi T., Miyagishi M., Taira K., Akira S., Fujita T. (2004). The RNA helicase RIG-I has an essential function in double-stranded RNA-induced innate antiviral responses. Nat. Immunol..

[20] Yoneyama M., Kikuchi M., Matsumoto K., Imaizumi T., Miyagishi M., Taira K., Foy E., Loo Y.M., Gale M., Akira S. (2005). Shared and unique functions of the DExD/H-box helicases RIG-I, MDA5, and LGP2 in antiviral innate immunity. J. Immunol..

[21] Kato H., Takeuchi O., Mikamo-Satoh E., Hirai R., Kawai T., Matsushita K., Hiiragi A., Dermody T.S., Fujita T., Akira S. (2008). Length-dependent recognition of double-stranded ribonucleic acids by retinoic acid-inducible gene-I and melanoma differentiation-associated gene 5. J. Exp. Med..

[22] Seth R.B., Sun L., Ea C.K., Chen Z.J. (2005). Identification and characterization of MAVS, a mitochondrial antiviral signaling protein that activates NF-κB and IRF 3. Cell.

[23] Potter J.A., Randall R.E., Taylor G.L. (2008). Crystal structure of human IPS-1/MAVS/VISA/Cardifcaspase activation recruitment domain. BMC Struct. Biol..

[24] Fitzgerald K.A., McWhirter S.M., Faia K.L., Rowe D.C., Latz E., Golenbock D.T., Coyle A.J., Liao S.M., Maniatis T. (2003). IKKepsilon and TBK1 are essential components of the IRF3 signaling pathway. Nat. Immunol..

[25] Takeuchi O., Akira S. (2008). MDA5/RIG-I and virus recognition. Curr. Opin. Immunol..

[26] Ohta A., Nishiyama Y. (2011). Mitochondria and viruses. Mitochondrion.

[27] Liang Y., Zhou Y., Shen P. (2004). NF-kappaB and its regulation on the immune system. Cell. Mol. Immunol..

[28] Oshiumi H., Okamoto M., Fujii K., Kawanishi T., Matsumoto M., Koike S., Seya T. (2011). The TLR3/TICAM-1 pathway is mandatory for innate immune responses to poliovirus infection. J. Immunol..

[29] Abe Y., Fujii K., Nagata N., Takeuchi O., Akira S., Oshiumi H., Matsumoto M., Seya T., Koike S. (2012). The toll-like receptor 3-mediated antiviral response is important for protection against poliovirus infection in poliovirus receptor transgenic mice. J. Virol..

[30] Richer M.J., Lavallee D.J., Shanina I., Horwitz M.S. (2009). Toll-like receptor 3 signaling on macrophages is required for survival following coxsackievirus B4 infection. PLoS ONE.

[31] Negishi H., Osawa T., Ogami K., Ouyang X., Sakaguchi S., Koshiba R., Yanai H., Seko Y., Shitara H., Bishop K. (2008). A critical link between Toll-like receptor 3 and type II interferon signaling pathways in antiviral innate immunity. Proc. Natl. Acad. Sci. USA.

[32] Hacker H., Redecke V., Blagoev B., Kratchmarova I., Hsu L.C., Wang G.G., Kamps M.P., Raz E., Wagner H., Hacker G. (2006). Specificity in Toll-like receptor signalling through distinct effector functions of TRAF3 and TRAF6. Nature.

[33] Stark G.R., Darnell J.E. (2012). The JAK-STAT pathway at twenty. Immunity.

[34] Ivashkiv L.B., Donlin L.T. (2014). Regulation of type I interferon responses. Nat. Rev. Immunol..

[35] Yamayoshi S., Yamashita Y., Li J., Hanagata N., Minowa T., Takemura T., Koike S. (2009). Scavenger receptor B2 is a cellular receptor for enterovirus 71. Nat. Med..

[36] Yang B., Chuang H., Yang K.D. (2009). Sialylatedglycans as receptor and inhibitor of enterovirus 71 infection to DLD-1 intestinal cells. Virol. J..

[37] Lin J.Y., Chen T.C., Weng K.F., Chang S.C., Chen L.L., Shih S.R. (2009). Viral and host proteins involved in picornavirus life cycle. J. Biomed. Sci..

[38] Kontsek P. (1994). Human type I interferons: Structure and function. ActaVirol..

[39] Parmar S., Platanias L.C. (2003). Interferons: Mechanisms of action and clinical applications. Curr. Opin. Oncol..

[40] Takaoka A., Yanai H. (2006). Interferon signalling network in innate defence. Cell. Microbiol..

[41] Sadler A.J., Williams B.R. (2008). Interferon-inducible antiviral effectors. Nat. Rev. Immunol..

[42] Yi L., He Y., Chen Y., Kung H.F., He M.L. (2011). Potent inhibition of human enterovirus 71 replication by type I interferon subtypes. Antivir. Ther..

[43] Liu M.L., Lee Y.P., Wang Y.F., Lei H.Y., Liu C.C., Wang S.M., Su I.J., Wang J.R., Yeh T.M., Chen S.H. (2005). Type I interferons protect mice against enterovirus 71 infection. J. Gen. Virol..

[44] Hung H.C., Wang H.C., Shih S.R., Teng I.F., Tseng C.P., Hsu J.T. (2011). Synergistic inhibition of enterovirus 71 replication by interferon and rupintrivir. J. Infect. Dis..

[45] Lu J., Yi L., Zhao J., Yu J., Chen Y., Lin M.C., Kung H.F., He M.L. (2012). Enterovirus 71 disrupts interferon signaling by reducing the level of interferon receptor 1. J. Virol..

[46] Kotenko S.V., Gallagher G., Baurin V.V., Lewis-Antes A., Shen M., Shah N.K., Langer J.A., Sheikh F., Dickensheets H., Donnelly R.P. (2003). IFN-lambdas mediate antiviral protection through a distinct class II cytokine receptor complex. Nat. Immunol..

[47] Ank N., West H., Bartholdy C., Eriksson K., Thomsen A.R., Paludan S.R. (2006). Lambda interferon (IFN-lambda), a type III IFN, is induced by viruses and IFNs and displays potent antiviral activity against select virus infections *in vivo*. J. Virol..

[48] Ank N., West H., Paludan S.R. (2006). IFN-lambda: Novel antiviral cytokines. J. Interferon Cytokine Res..

[49] Pott J., Mahlakoiv T., Mordstein M., Duerr C.U., Michiels T., Stockinger S., Staeheli P., Hornef M.W. (2011). IFN-lambda determines the intestinal epithelial antiviral host defense. Proc. Natl. Acad. Sci. USA.

[50] Lind K., Svedin E., Utorova R., Stone V.M., Flodstrom-Tullberg M. (2014). Type III interferons are expressed by Coxsackievirus-infected human primary hepatocytes and regulate hepatocyte permissiveness to infection. Clin. Exp. Immunol..

[51] Lind K., Richardson S.J., Leete P., Morgan N.G., Korsgren O., Flodstrom-Tullberg M. (2013). Induction of an antiviral state and attenuated coxsackievirus replication in type III interferon-treated primary human pancreatic islets. J. Virol..

[52] Cui S., Wang J., Fan T., Qin B., Guo L., Lei X., Wang J., Wang M., Jin Q. (2011). Crystal structure of human enterovirus 71 3C protease. J. Mol. Biol..

[53] Shih S.R., Chiang C., Chen T.C., Wu C.N., Hsu J.T., Lee J.C., Hwang M.J., Li M.L., Chen G.W., Ho M.S. (2004). Mutations at KFRDI and VGK domains of enterovirus 71 3C protease affect its RNA binding and proteolytic activities. J. Biomed. Sci..

[54] Sim A.C., Luhur A., Tan T.M., Chow V.T., Poh C.L. (2005). RNA interference against enterovirus 71 infection. Virology.

[55] Cai Q., Yameen M., Liu W., Gao Z., Li Y., Peng X., Cai Y., Wu C., Zheng Q., Li J. (2013). Conformational plasticity of the 2A proteinase from enterovirus 71. J. Virol..

[56] Kuo R.L., Kao L.T., Lin S.J., Wang R.Y., Shih S.R. (2013). MDA5 plays a crucial role in enterovirus 71 RNA-mediated IRF3 activation. PLoS ONE.

[57] Wang J.P., Cerny A., Asher D.R., Kurt-Jones E.A., Bronson R.T., Finberg R.W. (2010). MDA5 and MAVS mediate type I interferon responses to coxsackie B virus. J. Virol..

[58] Huhn M.H., McCartney S.A., Lind K., Svedin E., Colonna M., Flodstrom-Tullberg M. (2010). Melanoma differentiation-associated protein-5 (MDA-5) limits early viral replication but is not essential for the induction of type 1 interferons after Coxsackievirus infection. Virology.

[59] Barral P.M., Morrison J.M., Drahos J., Gupta P., Sarkar D., Fisher P.B., Racaniello V.R. (2007). MDA-5 is cleaved in poliovirus-infected cells. J. Virol..

[60] Barral P.M., Sarkar D., Fisher P.B., Racaniello V.R. (2009). RIG-I is cleaved during picornavirus infection. Virology.

[61] Lei X., Liu X., Ma Y., Sun Z., Yang Y., Jin Q., He B., Wang J. (2010). The 3C protein of enterovirus 71 inhibits retinoid acid-inducible gene I-mediated interferon regulatory factor 3 activation and type I interferon responses. J. Virol..

[62] Slater L., Bartlett N.W., Haas J.J., Zhu J., Message S.D., Walton R.P., Sykes A., Dahdaleh S., Clarke D.L., Belvisi M.G. (2010). Co-ordinated role of TLR3, RIG-I and MDA5 in the innate response to rhinovirus in bronchial epithelium. PLoSPathog..

[63] Lei X., Sun Z., Liu X., Jin Q., He B., Wang J. (2011). Cleavage of the adaptor protein TRIF by enterovirus 71 3C inhibits antiviral responses mediated by Toll-like receptor 3. J. Virol..

[64] Lei X., Xiao X., Xue Q., Jin Q., He B., Wang J. (2013). Cleavage of interferon regulatory factor 7 by enterovirus 71 3C suppresses cellular responses. J. Virol..

[65] Kawai T., Akira S. (2006). Innate immune recognition of viral infection. Nat. Immunol..

[66] Ning S., Pagano J.S., Barber G.N. (2011). IRF7: Activation, regulation, modification and function. Genes Immun..

[67] Akira S., Uematsu S., Takeuchi O. (2006). Pathogen recognition and innate immunity. Cell.

[68] Besse A., Lamothe B., Campos A.D., Webster W.K., Maddineni U., Lin S.C., Wu H., Darnay B.G. (2007). TAK1-dependent signaling requires functional interaction with TAB2/TAB3. J. Biol. Chem..

[69] Sato S., Sanjo H., Takeda K., Ninomiya-Tsuji J., Yamamoto M., Kawai T., Matsumoto K., Takeuchi O., Akira S. (2005). Essential function for the kinase TAK1 in innate and adaptive immune responses. Nat. Immunol..

[70] Lei X., Han N., Xiao X., Jin Q., He B., Wang J. (2014). Enterovirus 71 3C inhibits cytokine expression through cleavage of the TAK1/TAB1/TAB2/TAB3 complex. J. Virol..

[71] Takagaki Y., Manley J.L., MacDonald C.C., Wilusz J., Shenk T. (1990). A multisubunit factor, CstF, is required for polyadenylation of mammalian pre-mRNAs. Genes Dev..

[72] Weng K.F., Li M.L., Hung C.T., Shih S.R. (2009). Enterovirus 71 3C protease cleaves a novel target CstF-64 and inhibits cellular polyadenylation. PLoSPathog..

[73] Noah D.L., Twu K.Y., Krug R.M. (2003). Cellular antiviral responses against influenza A virus are countered at the posttranscriptional level by the viral NS1A protein via its binding to a cellular protein required for the 3′ end processing of cellular pre-mRNAS. Virology.

[74] Tsitsiou E., Lindsay M.A. (2009). microRNAs and the immune response. Curr. Opin. Pharmacol..

[75] Lui Y.L., Tan T.L., Woo W.H., Timms P., Hafner L.M., Tan K.H., Tan E.L. (2014). Enterovirus71 (EV71) utilise host microRNAs to mediate host immune system enhancing survival during infection. PLoS ONE.

[76] Ho B.C., Yu S.L., Chen J.J., Chang S.Y., Yan B.S., Hong Q.S., Singh S., Kao C.L., Chen H.Y., Su K.Y. (2011). Enterovirus-induced miR-141 contributes to shutoff of host protein translation by targeting the translation initiation factor eIF4E. Cell Host Microbe.

[77] Sukhatme V.P., Cao X.M., Chang L.C., Tsai-Morris C.H., Stamenkovich D., Ferreira P.C., Cohen D.R., Edwards S.A., Shows T.B., Curran T. (1988). A zinc finger-encoding gene coregulated with c-fos during growth and differentiation, and after cellular depolarization. Cell.

[78] Ho B.C., Yu I.S., Lu L.F., Rudensky A., Chen H.Y., Tsai C.W., Chang Y.L., Wu C.T., Chang L.Y., Shih S.R. (2014). Inhibition of miR-146a prevents enterovirus-induced death by restoring the production of type I interferon. Nat. Commun..

[79] Xu C., He X., Zheng Z., Zhang Z., Wei C., Guan K., Hou L., Zhang B., Zhu L., Cao Y. (2014). Downregulation of microRNA miR-526a by enterovirus inhibits RIG-I-dependent innate immune response. J. Virol..

[80] Oberste M.S., Penaranda S., Maher K., Pallansch M.A. (2004). Complete genome sequences of all members of the species Human enterovirus A. J. Gen. Virol..

[81] Kuo R.L., Kung S.H., Hsu Y.Y., Liu W.T. (2002). Infection with enterovirus 71 or expression of its 2A protease induces apoptotic cell death. J. Gen. Virol..

[82] Lin R., Lacoste J., Nakhaei P., Sun Q., Yang L., Paz S., Wilkinson P., Julkunen I., Vitour D., Meurs E. (2006). Dissociation of a MAVS/IPS-1/VISA/Cardif-IKKepsilon molecular complex from the mitochondrial outer membrane by hepatitis C virus NS3-4A proteolytic cleavage. J. Virol..

[83] Meylan E., Curran J., Hofmann K., Moradpour D., Binder M., Bartenschlager R., Tschopp J. (2005). Cardif is an adaptor protein in the RIG-I antiviral pathway and is targeted by hepatitis C virus. Nature.

[84] Yang Y., Liang Y., Qu L., Chen Z., Yi M., Li K., Lemon S.M. (2007). Disruption of innate immunity due to mitochondrial targeting of a picornaviral protease precursor. Proc. Natl. Acad. Sci. USA.

[85] Drahos J., Racaniello V.R. (2009). Cleavage of IPS-1 in cells infected with human rhinovirus. J. Virol..

[86] Mukherjee A., Morosky S.A., Delorme-Axford E., Dybdahl-Sissoko N., Oberste M.S., Wang T., Coyne C.B. (2011). The coxsackievirus B 3C protease cleaves MAVS and TRIF to attenuate host type I interferon and apoptotic signaling. PLoSPathog..

[87] Wang B., Xi X., Lei X., Zhang X., Cui S., Wang J., Jin Q., Zhao Z. (2013). Enterovirus 71 protease 2Apro targets MAVS to inhibit anti-viral type I interferon responses. PLoSPathog..

[88] Triantafilou K., Vakakis E., Kar S., Richer E., Evans G.L., Triantafilou M. (2012). Visualisation of direct interaction of MDA5 and the dsRNA replicative intermediate form of positive strand RNA viruses. J. Cell Sci..

[89] Feng Q., Langereis M.A., Lork M., Nguyen M., Hato S.V., Lanke K., Emdad L., Bhoopathi P., Fisher P.B., Lloyd R.E. (2014). Enterovirus 2Apro targets MDA5 and MAVS in infected cells. J. Virol..

[90] Morrison J.M., Racaniello V.R. (2009). Proteinase 2Apro is essential for enterovirus replication in type I interferon-treated cells. J. Virol..

[91] Hsu A.C., Parsons K., Barr I., Lowther S., Middleton D., Hansbro P.M., Wark P.A. (2012). Critical role of constitutive type I interferon response in bronchial epithelial cell to influenza infection. PLoS ONE.

[92] Liu Y., Zhang Z., Zhao X., Yu R., Zhang X., Wu S., Liu J., Chi X., Song X., Fu L. (2014). Enterovirus 71 inhibits cellular type I interferon signaling by downregulating JAK1 protein expression. Viral Immunol..

[93] Bovee M.L., Marissen W.E., Zamora M., Lloyd R.E. (1998). The predominant elF4G-specific cleavage activity in poliovirus-infected HeLa cells is distinct from 2A protease. Virology.

[94] Sommergruber W., Ahorn H., Klump H., Seipelt J., Zoephel A., Fessl F., Krystek E., Blaas D., Kuechler E., Liebig H.D. (1994). 2A proteinases of coxsackie- and rhinovirus cleave peptides derived from eIF-4 gamma via a common recognition motif. Virology.

[95] Haghighat A., Svitkin Y., Novoa I., Kuechler E., Skern T., Sonenberg N. (1996). The eIF4G-eIF4E complex is the target for direct cleavage by the rhinovirus 2A proteinase. J. Virol..

[96] Baeuerle P.A., Baltimore D. (1996). NF-κB: Ten years after. Cell.

[97] Pahl H.L. (1999). Activators and target genes of Rel/NF-κB transcription factors. Oncogene.

[98] Hiscott J., Nguyen T.L., Arguello M., Nakhaei P., Paz S. (2006). Manipulation of the nuclear factor-κB pathway and the innate immune response by viruses. Oncogene.

[99] Zheng Z., Li H., Zhang Z., Meng J., Mao D., Bai B., Lu B., Mao P., Hu Q., Wang H. (2011). Enterovirus 71 2C protein inhibits TNF-α-mediated activation of NF-κB by suppressing IκB kinase β phosphorylation. J. Immunol..

[100] Zhang X., Song Z., Qin B., Zhang X., Chen L., Hu Y., Yuan Z. (2013). Rupintrivir is a promising candidate for treating severe cases of enterovirus-71 infection: Evaluation of antiviral efficacy in a murine infection model. Antivir. Res..

[101] Shang L., Xu M., Yin Z. (2013). Antiviral drug discovery for the treatment of enterovirus 71 infections. Antivir. Res..

[102] Thomas A., Laxton C., Rodman J., Myangar N., Horscroft N., Parkinson T. (2007). Investigating Toll-like receptor agonists for potential to treat hepatitis C virus infection. Antimicrob. Agents Chemother..

[103] Horsmans Y., Berg T., Desager J.P., Mueller T., Schott E., Fletcher S.P., Steffy K.R., Bauman L.A., Kerr B.M., Averett D.R. (2005). Isatoribine, an agonist of TLR7, reduces plasma virus concentration in chronic hepatitis C infection. Hepatology.

[104] Isogawa M., Robek M.D., Furuichi Y., Chisari F.V. (2005). Toll-like receptor signaling inhibits hepatitis B virus replication *in vivo*. J. Virol..

[105] Lee J., Wu C.C., Lee K.J., Chuang T.H., Katakura K., Liu Y.T., Chan M., Tawatao R., Chung M., Shen C. (2006). Activation of anti-hepatitis C virus responses via Toll-like receptor 7. Proc. Natl. Acad. Sci. USA.

[106] Hammerbeck D.M., Burleson G.R., Schuller C.J., Vasilakos J.P., Tomai M., Egging E., Cochran F.R., Woulfe S., Miller R.L. (2007). Administration of a dual toll-like receptor 7 and toll-like receptor 8 agonist protects against influenza in rats. Antivir. Res..

[107] Cluff C.W., Baldridge J.R., Stover A.G., Evans J.T., Johnson D.A., Lacy M.J., Clawson V.G., Yorgensen V.M., Johnson C.L., Livesay M.T. (2005). Synthetic toll-like receptor 4 agonists stimulate innate resistance to infectious challenge. Infect. Immun..

[108] Ichinohe T., Watanabe I., Ito S., Fujii H., Moriyama M., Tamura S., Takahashi H., Sawa H., Chiba J., Kurata T. (2005). Synthetic double-stranded RNA poly(I:C) combined with mucosal vaccine protects against influenza virus infection. J. Virol..

[109] Wong J.P., Christopher M.E., Salazar A.M., Dale R.M., Sun L.Q., Wang M. (2007). Nucleic acid-based antiviral drugs against seasonal and avian influenza viruses. Vaccine.

[110] Levy O., Suter E.E., Miller R.L., Wessels M.R. (2006). Unique efficacy of Toll-like receptor 8 agonists in activating human neonatal antigen-presenting cells. Blood.

[111] Hsiao H.B., Chou A.H., Lin S.I., Chen I.H., Lien S.P., Liu C.C., Chong P., Liu S.J. (2014). Toll-like receptor 9-mediated protection of enterovirus 71 infection in mice is due to the release of danger-associated molecular patterns. J. Virol..

[112] Forrest S., Lear Z., Herod M.R., Ryan M., Rowlands D.J., Stonehouse N.J. (2014). Inhibition of the foot-and-mouth disease virus subgenomic replicon by RNA aptamers. J. Gen. Virol..

[113] Woo H.M., Kim K.S., Lee J.M., Shim H.S., Cho S.J., Lee W.K., Ko H.W., Keum Y.S., Kim S.Y., Pathinayake P. (2013). Single-stranded DNA aptamer that specifically binds to the influenza virus NS1 protein suppresses interferon antagonism. Antivir. Res..

[114] Ida-Hosonuma M., Iwasaki T., Yoshikawa T., Nagata N., Sato Y., Sata T., Yoneyama M., Fujita T., Taya C., Yonekawa H. (2005). The α/β interferon response controls tissue tropism and pathogenicity of poliovirus. J. Virol..

[115] Wessely R., Klingel K., Knowlton K.U., Kandolf R. (2001). Cardioselective infection with coxsackievirus B3 requires intact type I interferon signaling: Implications for mortality and early viral replication. Circulation.

